# Don’t delay, but don’t despair: symptom duration, comorbidity and outcome after closure of spinal cerebrospinal fluid leaks

**DOI:** 10.1007/s00415-024-12242-2

**Published:** 2024-02-26

**Authors:** Florian Volz, Amir El Rahal, Christian Fung, Mukesch Shah, Niklas Lützen, Horst Urbach, Jürgen Beck, Katharina Wolf

**Affiliations:** 1 Department of Neurosurgery, Medical Center, University of Freiburg, Breisacher Strasse 64, 79106 Freiburg, Germany; 2 Faculty of Medicine, University of Geneva, Geneva, Switzerland; 3 Department of Neurosurgery, Lindenhofspital, Bern, Switzerland; 4 Department of Neuroradiology, Medical Center, University of Freiburg, Freiburg, Germany

**Keywords:** Spinal cerebrospinal fluid leak, Spontaneous intracranial hypotension, Quality of life, Orthostatic headache, Symptom duration, Comorbidity

## Abstract

**Objective:**

Microsurgical sealing of spinal cerebrospinal fluid (CSF) leaks is a viable treatment option in spontaneous intracranial hypotension (SIH). Several factors may influence the outcome, with symptom duration probably the most modifiable variable.

**Methods:**

Patients with closure of spinal CSF leaks between September 2020 and March 2023 and a follow-up period of 6 months were included in this retrospective single-center study. Pre- and postoperative scores for impact of headaches (Headache Impact Test, HIT-6) and quality of life (QoL, EQ-5D-5L) were systematically collected. Multiple regression modelling and subgroup analyses for different symptom durations and comorbidities were performed for these outcomes.

**Results:**

One hundred patients (61% female, median age 43.5 years) were included. Six months postoperatively, there was significant improvement in headache impact (HIT-6: 66 (IQR 62–69) to 52 (IQR 40–61, *p* < 0.001) and QoL (EQ-5D-5L VAS: 40 (IQR 30–60) to 79 (IQR 60–90); EQ-5D-5L Index: 0.67 (IQR 0.35–0.8) to 0.91 (IQR 0.8–0.94, *p* < 0.001, respectively). Subgroup analysis for a symptom duration above (74%) and below 90 days (26%) and comorbidity, as well as multiple regression analysis, revealed a trend in favor of early treatment and lower comorbidity. However, even after a prolonged symptom duration, improvements were significant.

**Conclusion:**

As patients with shorter symptom duration show a trend for a better outcome, our results promote a timely diagnosis and treatment in SIH patients. However, a significant postoperative improvement can still be expected even after a prolonged symptom duration.

## Introduction

The clinical presentation of spontaneous intracranial hypotension (SIH) is multifaceted and includes, among many other symptoms, headache, neck pain, nausea, tinnitus and cognitive symptoms [1–4]. Typically, there is an acute and well-remembered onset of clinical symptoms. However, besides the classic orthostatic headache with an acute onset, there are also subtle and rather slowly progressing symptoms like position-independent headaches, cochlear disturbances and even late sequelae such as bibrachial amyotrophy or superficial siderosis [5, 6].

The primary cause of SIH are spontaneous spinal cerebrospinal fluid (CSF) leaks, which can be classified into different types [7–10]: Type 1 leaks (ventral leaks) are dural tears often associated with discogenic microspurs, usually at the disc level ventral to the spinal cord; Type 2 leaks (lateral leaks) are lateral dural tears in the area of a spinal nerve root; Type 3 leaks are CSF-venous fistulas. Recently, sacral dural tears have been identified as another cause of SIH [10]. After failure of conservative measures, sometimes including one or several epidural blood or fibrin glue patches, microsurgical closure is a viable option for all types of spinal CSF leaks [2], resulting in significant improvement of impact of headaches [9, 11–13] and quality of life [14] (Volz et al., Neurology: Clinical Practice, accepted 01/2024, CPJ-2023-000465). For CSF-venous fistulas, transvenous embolization [15, 16] and CT-guided fibrin glue occlusion [17, 18] also significantly improve clinical outcomes.

The time to diagnosis and adequate treatment can differ greatly and may be significantly delayed in some cases [1, 3]. The first study that found a significant effect of the symptom duration on the postoperative outcome after surgery was by Häni et al. [9]. Since then, a cut-off value of below 3 months as a predictor for a positive surgical outcome is frequently cited [3, 4].

In contrast to the symptom duration as a modifiable variable, preexisting comorbidities might also influence the postoperative outcome. However, a comprehensive assessment of comorbidity's impact on surgical outcomes in SIH patients is lacking.

This study aims to investigate the association of symptom duration and comorbidity on the outcome after surgical closure of spinal CSF leaks in a large patient cohort with systematically collected outcome measures and a 6-month follow-up.

## Methods

This retrospective cohort study from a tertiary center used systematically collected “patient-reported outcome measures” (PROMs) in patients with surgical closure of a spinal CSF leak between September 2020 and March 2023. The study followed the STROBE guidelines [19], and the local Ethics Committee approved the study (22-1512-S1-retro). All patients provided informed consent for evaluating and publishing their PROM data.

### Inclusion criteria

- SIH, according to the International Classification of Headache Disorders (ICHD-3) [20].
- Surgical treatment of a confirmed spinal CSF leak.
- Acute (start within 1 day) to subacute (start within 1 month) onset of clinical symptoms with a clear recall of the start of symptoms.
- Postoperative follow-up of at least 6 months.

### Exclusion criteria

- No remembered onset of clinical symptomatology.
- Incidental diagnosis of spinal CSF loss without clinical symptomatology.
- No consent for the use of PROM data.
- No ability to complete the questionnaires.
- No reliable contact information for the automated follow-up.

### Patient-reported outcome measures

#### SCQ

The 13-item Self-administered Comorbidity Questionnaire (SCQ) examines comorbidity by self-estimation of patients without professional medical examination [21, 22]. For 13 groups of diseases, the patient states “yes” or “no” whether a medical condition is present (SCQ problem score), whether a specific treatment was already initiated (SCQ treatment score), and whether the condition subjectively restricts normal activity (SCQ limitation score). Every answer of “yes” results in one point, thus leading to a summary score between 0 and 39 (SCQ summary score).

#### HIT-6

The 6-item Headache Impact Test (HIT-6) assesses the impact of headaches on daily life, with possible scores between 36 and 78 points [23]. HIT-6 scores below 50 points indicate no or little impact of headache in daily life [11, 12, 23, 24].

#### EQ-5D-5L

The EuroQol five-dimension five-level questionnaire (EQ-5D-5L) measures the quality of life, regardless of the underlying disease [25, 26]. It includes a visual analogue scale (EQ-VAS) that rates the current condition between the best (100 points) and the worst (0 points) imaginable health. Additionally, the level of impairment in five dimensions can be converted into an index score (EQ-Index) between 1 and < 0, which evaluates the patient's overall health status with an EQ-Index of 1 representing unimpaired health. The EQ-Index is calculated using country-specific weightings to reflect geographic differences. At the time of the surgery, 82/100 patients lived in Germany; the rest came from different European countries. Accordingly, the German value set for calculating the EQ-Index was used for all cases.

All questionnaires were completed preoperatively (baseline) and 6 months after surgery via an automated follow-up system. In the event of a revision surgery, the date of the second intervention was defined as the reference point for the follow-up.

### Clinical and diagnostic parameters

The duration of symptoms before surgery was determined by the personal statements of the patients at admission. Patients’ characteristics like sex, age, Body Mass Index (BMI), nationality, type of spinal CSF leak and the perioperative course (including postoperative neurological deficits and the need for revision surgery) were analyzed retrospectively after systematic collection.

### Diagnostics and surgical treatment

All patients underwent the local diagnostic protocol [27]. Finally, the type and exact level of the spinal leak was confirmed by dynamic digital subtraction myelography, cone-beam CT, and/or dynamic CT myelography [27, 28]. A minimally invasive tubular unilateral approach was used for all spinal leaks (Volz et al., Operative Neurosurgery, accepted 11/2023, 10.1227/ons.0000000000001042). Ventral leaks were sealed transdurally with an intradural/extradural sandwich patching technique [13]. Lateral leaks were sealed via surgical fibrin-patch augmentation, occasionally with additional clipping or suturing. CSF-venous fistulas were sealed with thermocoagulation of the draining vein after intraoperative visualization by intrathecal fluorescein injection [29] and additional clipping of the nerve root. Initial confirmation of successful sealing of the leak was based on the intraoperative assessment of the responsible surgeon and a subjective and objective clinical improvement of clinical symptoms in the first days after surgery. Final confirmation of a successful closure was achieved via clinical assessment and via routinely performed MRI of the brain and spine 3 months postoperatively.

### Statistics

For statistical analyses, SPSS Statistics^®^ (IBM Corporation, IBM SPSS Statistics for Macintosh, Version 29.0, Armonk, NY, USA) was used. Continuous data are presented as median with interquartile range (IQR), while categorical data are reported as frequencies. Patient-reported outcomes (PROMs) related to headache impact (HIT-6) and quality of life (EQ-VAS and EQ-Index) were compared between baseline and 6 months using the Wilcoxon signed-rank test. Subsequently, subgroup analyses were performed based on symptom duration (</≥ 90 days) and split by the median of the SCQ summary score (≤/> 1), with comparisons conducted using the Mann–Whitney *U* test. The association of symptom duration and comorbidities measured by SCQ summary score, age, sex, BMI, height, and leak type with patient-reported outcome measures at baseline and 6 months was assessed using multiple regression models, respectively. Bootstrapping (sample size 1000) was employed to correct for optimism. Symptom duration was log-transformed due to right-skewed data. In subsequent regression models, variables demonstrating potential effects (*p* < 0.2) were retained. Model fitting was evaluated using an *F*-statistic in an analysis of variance (ANOVA). We anticipated an indicative moderate goodness-of-fit (adjusted *R*^2^ ≥ 0.13) for reporting [30]. Statistical significance was assumed at *p* < 0.05.

## Results

### Patient population

**Fig. 1:**
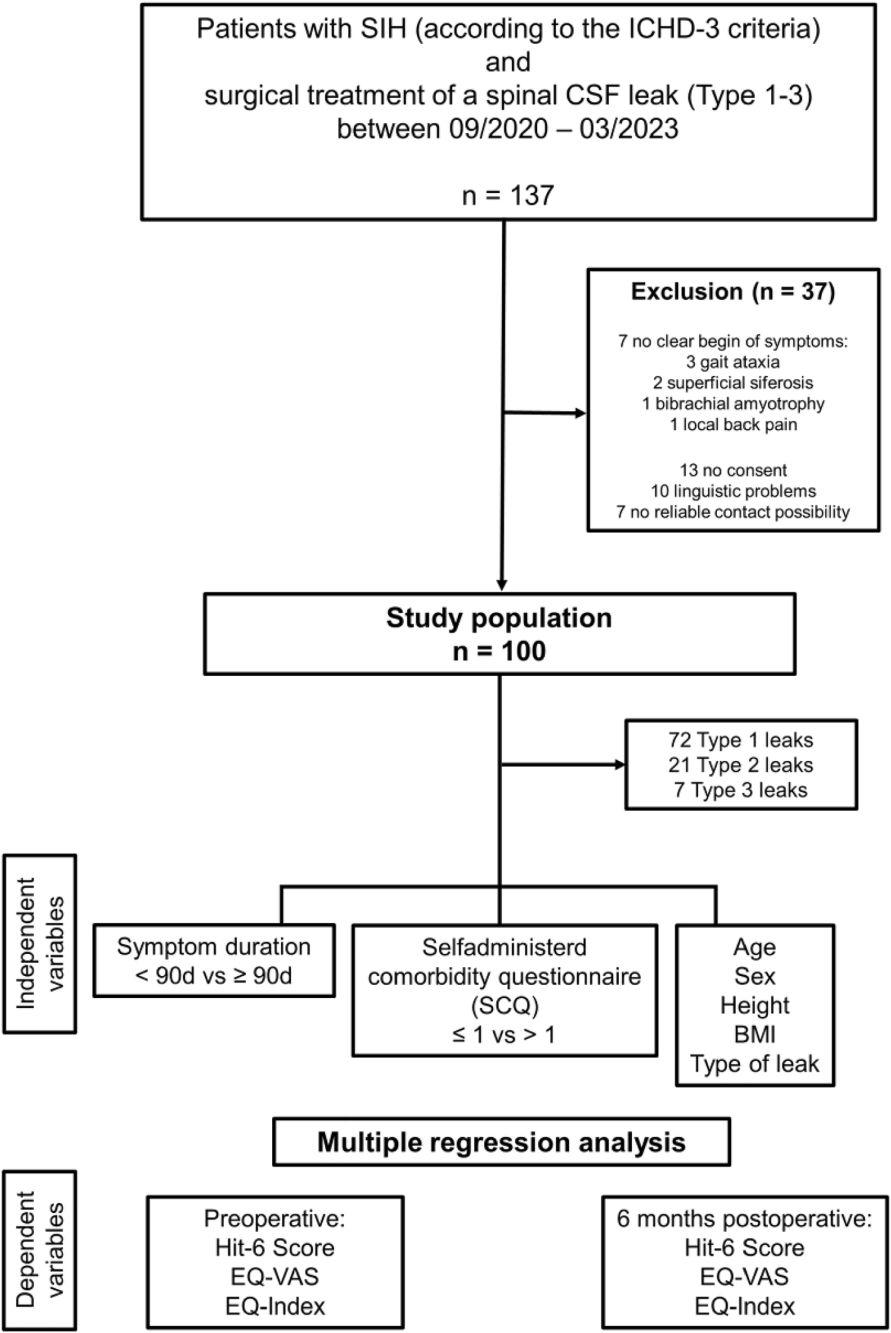
Flowchart displaying the patient selection and the parameters of the multiple regression analysis. Symptom duration and comorbidity (measured by the Self-Administered Comorbidity Questionnaire, SCQ) and demographic factors (age, sex, height, BMI and leak type) were considered independent variables. Results of the PROM scores preoperatively and 6 months postoperatively were considered as the dependent variables

Between September 2020 and March 2023, 137 patients underwent surgical closure of a spinal CSF leak in our institution. Among them, 30 were either technically or linguistically unable to complete the questionnaires or declined to do so. Additionally, seven patients could not recall a distinct onset of their symptoms; these individuals presented with gait ataxia (3/137), superficial siderosis (2/137), bibrachial amyotrophy (1/137), and localized back pain (1/137), thereby necessitating their exclusion. Consequently, the analysis included 100 patients (Fig. 1).

Of these patients, 61% were female. The median age was 43.5 years (IQR 36–53), and the median BMI was 23.9 kg/m^2^ (IQR 21.3–27.0). Leak classification revealed that 72% had ventral leaks, 21% had lateral leaks, and 7% had CSF-venous fistulas. The median duration of symptoms was 5 months (IQR 2–15), equivalent to 173 days (IQR 80–482), spanning a range from 9 days to 13 years. Regarding the SCQ summary score, the median was 1 (IQR 1–4), with scores ranging from 0 to 15. Notably, only 28 patients had a summary score of ≥ 4, and merely 2 had a score of ≥ 10 (Table 1).

**Table 1:** Patient characteristics

Patient characteristics	*N* = 100	
Female/male	61/39	
Type of spinal CSF leak		
Ventral leak (Type 1)	72	
Lateral leak (Type 2)	21	
CSF-venous fistula (Type 3)	7	

	Median (IQR)	Range
Age (years)	43.5 (36–53)	24–78
Body Mass Index (BMI, kg/m^2^)	23.9 (21.3–27.0)	16.9–38.5
Duration of symptoms		
Months	5 (2–15.3)	0.3–157
Days	173 (80–482)	9–4792
Self-administered comorbidity questionnaire (SCQ)		
SCQ—Problem Score	1 (0–2)	0–6
SCQ—Treatment Score	0 (0–1)	0–5
SCQ—Limitation Score	0 (0–1)	0–4
SCQ—Summary Score	1 (0–4)	0–15

### Patient-reported outcome

**Fig. 2:**
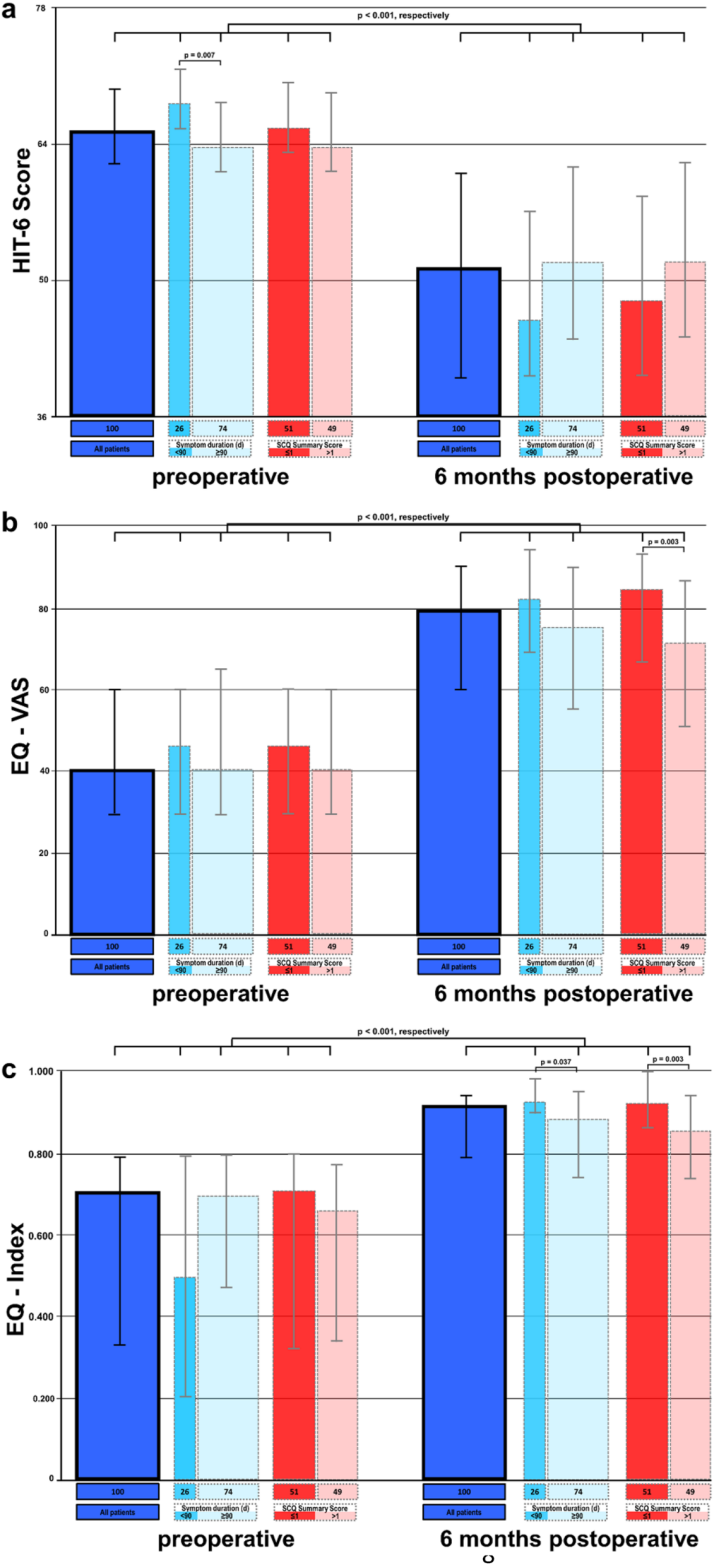
Bar graph of the results for the HIT-6 Score (**a**), the visual analogue scale of the EQ-5D-5L (EQ-VAS, **b**) and the EQ-5D-5L index score (EQ-Index, **c**) preoperatively and 6 months after surgery. The height of the bars represent the median; the whiskers represent the interquartile range (IQR) from the first to the third quartile. The dark blue bars represent the whole patient cohort, and the two light bars display the patient subgroups with a symptom duration of fewer than 90 days (more saturated blue) or higher (translucentblue), with the width of the bars representing the size of the subgroup. Accordingly, the two red bars display the patient subgroups with a SCQ summary score of ≤ 1 (darker red) or higher (lighter red). All scores significantly improved in the complete patient cohort and in all subgroups (*p* < 0.001, respectively). Preoperatively, patients with a shorter symptom duration experience a higher impact of headaches (*p* = 0.007) and reach a postoperative score of < 50 points, indicating no or only little impact of headaches. Patients with a shorter symptom duration reach a higher level of quality of life, with a significant difference in the EQ-Index (*p* = 0.037). Although both subgroups experience a clear improvement in quality of life after surgery, patients with higher comorbidity end up with significantly lower scores for EQ-VAS and EQ-Index

The median HIT-6 scores significantly improved 6 months after sealing of the CSF leaks from 65.5 (IQR 61.8–69.3) to 51.5 (IQR 40.0–61.0, *p* < 0.001). Both measurements for Quality of life (QoL) significantly increased: EQ-VAS increased from 40 (IQR 30–60) to 79 (IQR 60–90), and EQ-Index increased from 0.669 (IQR 0.345–0.799) to 0.910 (IQR 0.796–0.943) (*p* < 0.001, respectively) (Table 2, Fig. 2).

**Table 2:** Results for the HIT-6 Score, EQ-VAS, and EQ-Index preoperatively and 6 months after surgery

	*N*	Preoperative Median (IQR)	6 months postoperative Median (IQR)	*p* value
Headache Impact Test (HIT-6)				
All patients	100	65.5 (61.8–69.3)	51.5 (40.0–61.0)	< 0.001
Duration of symptoms < 90 days	26	68.5 (65.0–71.0)**	46.0 (40.0–57.5)	< 0.001
Duration of symptoms ≥ 90 days	74	64.0 (61.0–68.0)**	52.0 (44.0–61.8)	< 0.001
SCQ Summary Score ≤ 1	51	66.0 (63.0–70.0)	48.0 (40.0–58.5)	< 0.001
SCQ Summary Score > 1	49	64.0 (61.0–69.0)	52.0 (44.0–62.0)	< 0.001
EuroQol-5D-5L (EQ-5D-5L)				
EQ-VAS				
All patients	100	40 (30–60)	79 (60–90)	< 0.001
Duration of symptoms < 90 days	26	46 (30–60)	82 (69–94)	< 0.001
Duration of symptoms ≥ 90 days	74	40 (30–65)	75 (56–90)	< 0.001
SCQ Summary Score ≤ 1	51	46 (30–60)	84 (67–93)**	< 0.001
SCQ Summary Score > 1	49	40 (30–60)	71 (50–85)**	< 0.001
EQ-Index				
All patients	100	0.669 (0.345–0.799)	0.910 (0.796–0.943)	< 0.001
Duration of symptoms < 90 days	26	0.494 (0.210–0.793)	0.923 (0.909–0.992)*	< 0.001
Duration of symptoms ≥ 90 days	74	0.692 (0.480–0.799)	0.881 (0.737–0.943)*	< 0.001
SCQ Summary Score ≤ 1	51	0.704 (0.343–0.814)	0.918 (0.866–1.000)**	< 0.001
SCQ Summary Score > 1	49	0.656 (0.359–0.786)	0.851 (0.734–0.934)**	< 0.001

The whole patient cohort and all subgroups significantly improved in all scores (*p* < 0.001, respectively). Significant differences between subgroups are marked with *p < 0.05 and **p < 0.01

### The impact of symptom duration, comorbidity, and clinical parameters

Essentially, all patients, divided by symptom duration (</≥ 90 days) and divided by comorbidity (SCQ summary scores ≤/> 1), demonstrated a significant improvement in the impact of headaches and quality of life (*p* < 0.001, respectively, Table 2, Fig. 2).

Multiple regression analysis revealed a moderate correlation of longer symptom duration with reduced QoL 6 months after surgery (*p* = 0.020 for EQ-VAS and *p* = 0.035 for EQ-Index). Higher comorbidity scores moderately correlated with a more pronounced impact of headaches and reduced QoL 6 months after surgery (*p* = 0.011 for HIT-6, *p* = 0.001 for EQ-VAS and *p* = 0.006 for EQ-Index) (Table 3). However, no substantial effect of symptom duration was observed in the second component of the QoL measurement (EQ-VAS) or in the HIT-6 score, both at baseline and the 6-month follow-up. Male sex showed a moderate correlation with a worse perceived QoL at baseline (*p* = 0.004 for EQ-VAS and *p* = 0.003 for EQ-Index). Age, BMI and type of leak did not correlate with the outcome parameters.

**Table 3:** The effect of clinical and diagnostic parameters on outcome

	Goodness-of-fit			Symptom duration (ln) (days)	SCQ Summary Score	Sex (m < f)	Age (years)	BMI (kg/m^2^)	Height (cm)	Leak type
Preoperative										
HIT-6	*R*	0.234	*B*	− 1.1						
	Adj. *R*^2^	0.035	95% CI	− 2.2/0.2						
	*p*	0.065	*p*	0.081	> 0.1	> 0.1	> 0.1	> 0.1	> 0.1	> 0.1
EQ-VAS	*R*	0.400	*B*		− 1.9	− 19.3			− 87.9	− 4.7
	Adj. *R*^2^	0.096	95% CI		− 3.2/− 0.3	− 31.1/− 6.5			− 163.2/− 14.7	− 9.2/0.5
	*p*	0.021	*p*	> 0.1	**0.007**	**0.004**	> 0.1	> 0.1	**0.019**	0.065
EQ-Index	*R*	0.484	*B*			− 0.2				
	**Adj. *R***^**2**^	**0.176**	95% CI			− 0.4/− 0.1				
	*p*	0.001	*p*	> 0.1	> 0.1	**0.003**	> 0.1	> 0.1	> 0.1	> 0.1
6 months postoperative										
HIT-6	*R*	0.323	*B*		0.9		− 0.2	0.4		
	Adj. *R*^2^	0.076	95% CI		0.1/1.5		− 0.3/0.0	− 0.0/0.8		
	*p*	0.014	*p*	> 0.1	**0.011**	> 0.1	0.055	0.080	> 0.1	> 0.1
EQ-VAS	*R*	0.499	*B*	− 3.1	− 2.7					
	Adj. *R*^2^	**0.192**	95% CI	− 5.8/− 0.5	− 3.9/− 1.3					
	*p*	< 0.001	*p*	**0.020**	**0.001**	> 0.1	> 0.1	> 0.1	> 0.1	> 0.1
EQ-Index	*R*	0.475	*B*	− 0.03	− 0.03					
	**Adj. *R***^**2**^	**0.167**	95% CI	− 0.06/− 0.01	− 0.05/− 0.01					
	*p*	0.001	*p*	**0.035**	**0.006**	> 0.1	> 0.1	> 0.1	> 0.1	> 0.1

Results of multiple regression analysis are reported: *R*—correlation coefficient, adj—adjusted, *B*—regression coefficient. Bold lettering marks models indicating a moderate association of the results with the outcome measures (adj. *R*^2^ > 0.13)

In the subgroup analysis, patients with a symptom duration < 90 days reported higher baseline HIT-6 scores compared to those with longer lasting symptoms, with both groups experiencing severe impact of headaches (68.5 (IQR 65–71) vs. 64 (IQR 61–68), *p* = 0.007). However, while HIT-6 scores at the 6-month mark were not statistically different (46 (IQR 40–57.5) vs. 52 (IQR 44–61.8), *p* = 0.157), the improvement was higher in the patient subgroup with a shorter symptom duration. Patients with a symptom duration < 90 days exhibited a significantly better outcome in EQ-Index (0.923 (IQR 0.909–0.992) vs. 0.881 (IQR 0.737–0.943), *p* = 0.037).

Patients with a SCQ summary scores ≤ 1 had higher HIT-6 scores preoperatively (66 (IQR 63–70) vs. 64 (IQR 61–69), *p* = 0.08) and a more pronounced improvement after the surgery (48 (IQR 40–48.5) vs. 52 (IQR 44–62), *p* = 0.25), but without a statistically significant difference, respectively. Patients with SCQ summary scores > 1 had consistently lower EQ-VAS and EQ-Index scores preoperatively. Postoperatively, there was a significant overall improvement in both subgroups. However, patients with SCQ summary scores ≤ 1 reached significantly higher QoL scores after surgery (EQ-VAS 84 (IQR 67–93) vs. 71 (IQR 50–85), *p* = 0.003 and EQ-Index 0.918 (IQR 0.866–1.00) vs. 0.851 (IQR 0.734–0.934), *p* = 0.003).

### Complications

Except for expectable unilateral hypoesthesia in the respective dermatome in cases with clipping of a thoracic nerve root (3/21 lateral leaks and 7/7 cases of CSF-venous fistulas), no permanent neurological deficit occurred. Ten patients (10%, 6 ventral leaks and 4 lateral leaks) needed revision surgery: three patients (all ventral leaks) due to an epidural hematoseroma. Two patients (one ventral and one lateral leak) had inconclusive intraoperative findings; the subsequent postoperative imaging revealed “wrong level” surgery. Five patients (two ventral and three lateral leaks) had no improvement or a recurrence of their original symptoms; the subsequent imaging revealed a recurrence of the original leak within 10 days up to 5 months.

## Discussion

This study confirms previous results of the significant and long-lasting improvement of the impact of headaches and quality of life (QoL) after surgical closure of spinal CSF leaks in a large patient cohort using systematically collected patient-reported outcome measures (PROMs) with validated scores and a complete 6-month follow-up. All patients combined and all subgroups with a symptom duration of below or above 90 days, as well as lower or higher comorbidity, significantly benefited from surgical treatment with a moderate trend in favor of shorter symptom duration and lower comorbidity.

### Symptom duration and outcome

Symptom duration before a targeted treatment of spinal CSF leaks shows a high variability. Our study’s median symptom duration was 5 months, ranging between 9 days and 13 years. Cheema et al. report a median time from symptom onset to the correct diagnosis of 2 months with a range of 0–180 months, and after that, a median period of 10 weeks until surgery [3]. Before surgical treatment of CSF-venous fistulas, Wang et al. report an average duration of symptoms of around 40 months [11] and Lohkamp et al. a symptom duration between 1.5 and 3 years [31]. Notably, neither study reports an impact of symptom duration on the outcome. Häni et al. report a mean symptom duration of around 12 months before surgical treatment [9] of different leak types. In studies of patients with CSF-venous fistulas, Brinjikji et al. report a mean symptom duration of 40.9 months before transvenous embolization [16] and Callen et al. a mean symptom duration of 34.6 months before CT-guided fibrin glue occlusion [18].

The latter three studies report a significant impact of symptom duration on the postoperative outcome. In Häni’s series, the duration is the only factor with a significant impact. They report, that a symptom duration below 87 days was correlated with a better outcome [9], promoting the recommendation to strive for a prompt diagnosis and therapy, ideally within 3 months if SIH is suspected [3, 4]. In Brinjikji’s series, several factors influence the outcome, with symptom duration as the only modifiable variable [16]. Callen et al. found, among other variables, a significant association between symptom duration and the clinical improvement [18]. However, in all three studies, the clinical outcome was analyzed using three-step ordinal scales without specific scores for the impact of headaches or QoL.

Our study correlated the symptom duration and the level of comorbidities among other clinical parameters using validated patient-reported outcome measures (PROMs) for the impact of headaches on daily life (via HIT-6) and quality of life (QoL via EQ-VAS and EQ-Index), allowing for a more nuanced description and interpretation. Prolonged symptom duration and increased comorbidities moderately correlated with reduced QoL. Therefore, prompt treatment to avoid prolonged disease duration is recommended, as it is the only modifiable variable. However, it is important to note that our results pertain to a natural logarithmic scale of disease duration. This implies that higher levels of change occur in the early phase of the disease, with diminishing effects in later stages. In simpler terms, our findings suggest that postponing treatment to 1 or 2 years from symptom onset, instead of intervening at 90 days, could potentially reduce EQ-VAS scores by about 4 to 6.5 points, respectively. Conversely, early intervention, such as at 10 days, could lead to an additional improvement of 7 points compared to surgery at 90 days (Table 3). Given the wide confidence intervals and the predominance of patients with a disease duration of more than 90 days, caution is necessary in interpreting these approximations, and further studies are necessary to confirm the results.

HIT-6 scores at baseline were significantly higher in patients with shorter symptom duration, representing a more severe impact of headaches. This observation aligns with the commonly reported phenomenon where orthostatic headaches, characteristic at the onset of the disease, may become less typical and recede into the background during a prolonged course [1, 20, 32–34]. The subgroup with shorter symptom duration demonstrated a higher improvement in HIT-6 scores of 22.5 points 6 months after surgery (baseline 68.5 to 46 at 6 months). Notably, the postoperative score of 46 is clearly below the widely recognized threshold of 50 points, indicating no or minimal impact of headaches on daily life [11, 12, 23, 24]. Patients with a symptom duration exceeding 90 days, while still benefitting significantly from surgery, did not reach this decisive cut-off (median HIT-6 score: baseline 64 to 52 at 6 months). Possible explanations for these findings may include the chronicity of headaches, a potential association with anxiety or depression after a prolonged course, a potential overlap with different headache types, and extended readaptation processes of CSF and venous homeostasis. However, the observed improvement of 12 points in the HIT-6 Score is undeniably significant and clinically relevant [12, 35, 36]. These results reinforce the importance of prompt diagnosis and appropriate therapy, while longer symptom duration does not preclude a significant headache improvement.

### Comorbidity assessments in SIH patients

This study marks the first systematic investigation into comorbidities among surgically treated SIH patients using a well-established scoring system. The Self-administered Comorbidity Questionnaire (SCQ) efficiently integrates patient-reported data into a systematic Patient-Reported Outcome Measure (PROM) tool, sidestepping potential issues with incomplete datasets seen in medical record-based assessments. This method provides reliable results without relying on medical professionals’ records. Various studies have highlighted the SCQ’s predictive power, likening it to the commonly used Charlson Comorbidity Index [21, 22, 37]. Obviously, pre-existing comorbidity stands as an inherent non-modifiable factor before any surgical treatment. Our study demonstrates that comorbidity is relatively low in a representative cohort of SIH patients. 50% exhibited no comorbidity, with only 2% exhibiting a SCQ score above 10. This reaffirms previous claims about the relevance of the disease, which suddenly severely affects otherwise completely healthy patients in their everyday lives [3, 38] (Volz et al., Neurology Clinical Practice, accepted, CPJ-2023-000465R2). However, this low comorbidity contributes decisively to the markedly positive benefit–risk profile and the swift, substantial postoperative improvement.

Corresponding to the findings regarding symptom duration, patients with lower comorbidity initially present with higher preoperative HIT-6 scores, yet they achieve better improvement, reaching a median score below 50 points, indicating minimal or no impact of headaches. However, these trends, while suggestive, do not reach statistical significance. Notably, the subgroup with higher comorbidity consistently displays lower QoL scores. While both subgroups significantly benefit from surgery, patients without comorbidity reach an even better outcome.

## Limitations

The limitations of a specialized tertiary single-center series with a highly selected patient cohort apply to this study. However, our research benefits from systematically collected patient-reported outcome measures (PROMs) utilizing validated and standardized scores, together with a comprehensive 6-month follow-up period. As the association between radiological findings, particularly the Bern SIH Score [39], and the clinical outcome still needs to be completely understood, we deliberately emphasize the clinical perspective and do not include radiological features in our statistical analysis.

## Conclusions

Six months after surgical closure of spinal CSF leaks, the impact of headaches and quality of life are significantly improved. This improvement is higher when the symptom duration is < 90 days. Comorbidity is generally low in the SIH population, with a higher comorbidity associated with more impaired QoL before and after surgical treatment. Our results further promote the recommendation for a timely diagnosis and treatment in SIH patients. However, even after a prolonged symptom duration, a significant improvement can still be expected, and surgical therapy should not be withheld from these patients. In short, don’t delay a swift surgical treatment, but don’t despair in cases with a longer symptom duration.

## Data Availability

The study data are shared at reasonable request from other investigators.

## Abbreviations

CSF: Cerebrospinal fluid
EQ-5D-5L: EuroQol five-dimension five-level questionnaire
EQ-Index: Index Score of the EQ-5D-5L
EQ-VAS: Visual analogue scale of the EQ-5D-5L
HIT-6: 6-Item Headache Impact Test
PROMs: Patient-reported outcome measures
QoL: Quality of life
SCQ: 13-Item Self-Administered Comorbidity Questionnaire
SIH: Spontaneous intracranial hypotension
